# Investigating the Reductive Phosphatization Reaction Pathway in the Synthesis of Transition Metal Phosphates: A Case Study on Titanium Phosphates

**DOI:** 10.1021/acs.inorgchem.4c04776

**Published:** 2025-01-24

**Authors:** Hilke Petersen, Niklas Stegmann, Wolfgang Schmidt, Claudia Weidenthaler

**Affiliations:** Heterogeneous Catalysis, Max-Planck-Institut für Kohlenforschung, Kaiser-Wilhelm-Platz 1, Mülheim 45470, Germany

## Abstract

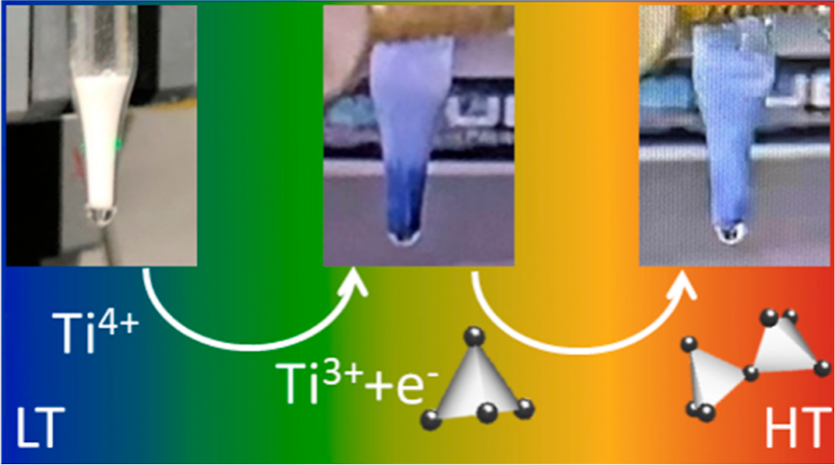

Reductive phosphatization is an original synthesis approach to the formation of transition metal phosphates (TMPs). The approach enables the synthesis of known TMPs, but also new compounds, especially with transition metals in a low-valent state. However, to exploit the enormous potential of this synthesis method, it is necessary to identify and characterize all of the potential intermediates and final synthesis products. Here, we report on in situ synchrotron X-ray powder diffraction experiments to unravel the temperature-dependent formation pathway of TMPs using TiO_2_–NH_4_H_2_PO_2_ as an example. The pathway consists of several consecutive steps, including the melting of NH_4_H_2_PO_2_, which acts as a reducing agent and a reaction medium. A reduction in the ratio of TiO_2_ to NH_4_H_2_PO_2_ decelerates the reaction and causes increased impurity formation. The hypophosphite melt reduces Ti^4+^ in TiO_2_ to Ti^3+^, and a previously unknown compound, denoted as **Ti(III)po** with chemical composition (NH_4_)_*x*_H_1–*x*_Ti(HPO_4_)_2_, is formed. In a subsequent step, (NH_4_)_*x*_H_1–*x*_Ti(HPO_4_)_2_ reacts in a polycondensation reaction to form monoclinic NH_4_TiP_2_O_7_, denoted as **Ti(III)p** in our earlier work.

## Abstract 2

Reductive phosphatization enables the synthesis of TMPs, including new low-valent compounds. To predict the synthesis products, an understanding of the reaction mechanism is essential. Using in situ synchrotron powder diffraction, we have studied TiO_2_–NH_4_H_2_PO_2_ as an example. The mechanism involves the melting of NH_4_H_2_PO_2_, and reduction of TiO_2_ to form (NH_4_)_*x*_H_1–*x*_Ti(HPO_4_)_2_ Ti(III)(NH4)x(H1-xPO4)_2_, which then undergoes polycondensation to NH_4_TiP_2_O_7_.

## Introduction

Transition metal phosphates (TMPs) can have a wide range of chemical compositions, resulting in a variety of structural motifs with different metal coordinations and variations of the phosphate units. The phosphate structures can be divided into (a) orthophosphates with isolated [PO_4_]^3–^ units, (b) pyrophosphates with two connected units forming a dimer [P_2_O_7_]^4–^, (c) cyclic metaphosphates with [P_*n*_O_3*n*_]^*n*−^ motifs, and (d) linear polyphosphates with chains built of [PO_3_]_*n*_^*n*–^ polyhedra. The wide range of possible chemical compositions, different crystal structures, and properties open up a multitude of potential applications. One example is the use of the orthophosphate LiFePO_4_ as an efficient and environmentally sustainable cathode material in energy storage. The structure determines the performance of LiFePO_4_ as a cathode material, particularly for rechargeable batteries.^1−7^ LiFePO_4_ combines the advantages of high rate capability as well as good reversibility with environmental sustainability and low cost.^1,4,6,8−10^ Another famous TMP is the commercially used VO(P_2_O_7_) catalyst for the selective oxidation of butane to maleic anhydride.^11−14^*Operando* and in situ Raman studies revealed a complex activation process of the precatalyst VOHPO_4_·0.5H_2_O to the active catalyst phase VO(P_2_O_7_). This includes multiple reorganization processes on the crystallite surface as well as of the crystalline bulk material.^11,12^ Recently, it has been reported that titanium phosphates also exhibit the rare ability to perform this demanding selective partial oxidation reaction.^15^ The family of titanium phosphates is among the most intensively studied TMPs, as they are promising candidates for catalysis and/or photocatalysis, as solid acids, or as proton conductors. One acid catalyst is the tetragonal orthophosphate Ti(HPO_4_)_2_·H_2_O, which crystallizes in *I*4_1_/*a* with lattice parameters *a* = 6.335 Å and *c* = 16.389 Å. In the structure, each titanium atom is connected to six orthophosphate groups, forming channels along the crystallographic *c*-axis. The hydroxide groups are pointing in the cavities, creating acid sites active for catalysis.^16^ Pyrophosphates with the chemical composition of **MP**_**2**_**O**_**7**_ with M = Sn, Ti, Si, Ge, Zr, and Ce show potential as proton-exchange membrane materials for next-generation fuel cells, operating in the intermediate temperature range.^2,17^ These compounds are particularly suitable for this application due to their proton conductivity between 273 and 673 K under anhydrous conditions.^2^ The structure of **MP**_**2**_**O**_**7**_ was originally described in the space group *Pa* 3̅ with a lattice parameter of about 8 Å (*Z* = 4) by Levi and Peyronel.^18^ However, several publications report a low-temperature superstructure for TiP_2_O_7_ in the space group *Pa* 3̅ with a 3 × 3 × 3 cell (*Z* = 108) and a lattice parameter of *a* = 23.6383(2) Å.^19,20^

The compositions and structures of TMPs are highly dependent on the precursors used and the method of synthesis. Various preparation methods are reported in the literature, such as hydrothermal, molten salt, and precipitation syntheses. All conventional methods use a transition metal and a phosphate compound as precursors.^21−26^ We have recently published a new synthesis approach for TMPs.^27^ In contrast to the conventional synthesis procedures, a molten hypophosphite is used both as a phosphate source and as a liquid reaction medium. The novelty of this process is that NH_4_H_2_PO_2_ is a strong reducing agent. Several TMPs known from the literature containing Ti, V, Cr, Mn, and Fe were synthesized by this method. However, the key feature of this synthesis procedure is the ability to control the oxidation state of the transition metal and form TMPs with the transition metal in a low oxidation state. As a result, a number of so far unknown compounds have been obtained.^27^ In the system TiO_2_–NH_4_H_2_PO_2_, reductive phosphatization in ambient atmosphere led to the formation of TiP_2_O_7_ with tetravalent titanium (*T* = 500 °C), while in an inert atmosphere, Ti(PO_3_)_3_ (*T* = 500 °C) and two so far unknown pyrophosphate phases containing trivalent titanium atoms could be obtained. The crystal structure of two pyrophosphates has been solved from powder diffraction data: a monoclinic compound with trivalent titanium and the formula NH_4_TiP_2_O_7_, denoted as **Ti(III)p**, and a triclinic one containing tetravalent titanium and the formula TiP_2_O_7_, denoted as **Ti(IV)p**.^28^ The monoclinic **Ti(III)p** crystallizes in the space group *P*2_1_/*c* with the metric parameters *a* = 7.5457(2) Å, *b* = 10.2550(2) Å, *c* = 8.2573(6) Å, and β = 105.925(6)°. The compound consists of a layered structure in which TiO_6_ octahedra are coordinated by five pyrophosphate units. The TiO_6_ octahedron is distorted with Ti–O distances ranging from 1.98(1) to 2.13(1) Å. Four pyrophosphate anions are bound as a monodentate ligand and one as a bidentate ligand. The local arrangement of the TiO_6_ and pyrophosphate units results in the formation of one-dimensional channels running along the crystallographic *c-*axis. These channels are stabilized by NH_4_^+^ cations. For **Ti(III)p**, good proton conductivity in the range of 10^–3^ S cm^–1^ with an activation energy of 0.17 eV was measured in the fully hydrated state. The activation energy is in good agreement with the Grotthus mechanism for the proton conductivity with the NH_4_^+^ cations supporting the proton transfer.^28^

**Ti(IV)p** forms by annealing **Ti(III)p** in inert atmospheres. The compound crystallizes in *P* 1̅ with *a* = 6.2292(1) Å, *b* = 7.9483(1) Å, *c* = 6.2065(1) Å, α = 102.804(2)°, β = 74.8277(18)°, and γ = 83.203(2)°. During the reaction, NH_4_^+^ ions decompose to NH_3_ and H_2_;^16^ the formation of the latter causes the oxidation of Ti^3+^ to Ti^4+^. The TiO_6_ octahedra become more regular with Ti–O distances of 1.90(1)–1.96(1) Å, but the local coordination by the pyrophosphate units remains almost unchanged.^28^

In our previous work, **Ti(III)p** and **Ti(IV)p** were synthesized in the batch mode in tube furnaces as the final products applying given reaction parameters. The question how the phases do form and whether they form upon heating or during cooling of the melt could not been addressed. In the present study, we now present in situ X-ray powder diffraction (XRPD) data that monitor the processes taking place during reductive melt synthesis from TiO_2_ and NH_4_H_2_PO_2_ leading in the formation of **Ti(III)p**. To unravel reaction pathways of this new synthesis approach, fast in situ diffraction measurements at synchrotrons are mandatory to monitor also the formation of possible intermediate states. The data provide important insights into the formation process and are fundamental for the targeted synthesis of the individual compounds.

## Experimental Methods

### X-ray Powder Diffraction

The XRPD patterns of the ex situ obtained samples were recorded on a Rigaku SmartLab diffractometer (Tokyo, Japan) equipped with a 9 kW rotating anode emitting Cu K_α1,2_ radiation. The measurements were carried out in the Bragg–Brentano mode with an incident slit of 0.08° and a 5 mm mask. The data were recorded with a step size of 0.01° 2θ and a scan rate of 1° min^–1^. The obtained data were analyzed using the DiffracPlus TopasV6 software (Bruker AXS GmbH, Karlsruhe, Germany).^29^ The simulated annealing and charge flipping approaches implemented in the software were used for the structure determination.

### Ex Situ Total Scattering Experiments and Subsequent Pair Distribution Function Analysis

The total scattering data for subsequent qualitative pair distribution function (PDF) analysis were recorded with a laboratory diffractometer (STOE STADIP) in a transmission geometry (MoKα_1_ 0.71073 Å) and a Mythen-1D detector. Subsequently, the PDF data were generated with the *PDFgetX3* software (Columbia University, New York, NY).^30^ The *Q*_maxinstr_ was 16 Å^–1^, and the *Q*_max_ of the evaluated data was 15 Å^–1^. As the data quality and resolution in *Q* are limited, the data were only used for the identification of interatomic distances but not for the refinement of the local structure.

### In Situ XRPD Measurements Performed During the Synthesis

The in situ XRPD experiments were carried out at beamline P02.1 (PETRA III DESY, Hamburg, Germany). The reaction mixtures were heated using the mini hot air blower provided by beamline P02.1. The data were collected in the temperature range between 30 and 320 °C (heating rate 20 K min^–1^) with a Varex XRD 4343CT detector using a wavelength of 0.20734 Å and a counting rate of 10 s per frame. For data quality, data from six frames were summed before integration. Data integration was performed with the software package Dawn 2.6.0 (Diamond Light Source Ltd., Oxfordshire, United Kingdom),^31^ XRPD data were analyzed using the *DiffracPlus TopasV6* software (Bruker AXS GmbH, Karlsruhe, Germany).^29^

**Figure 1:**
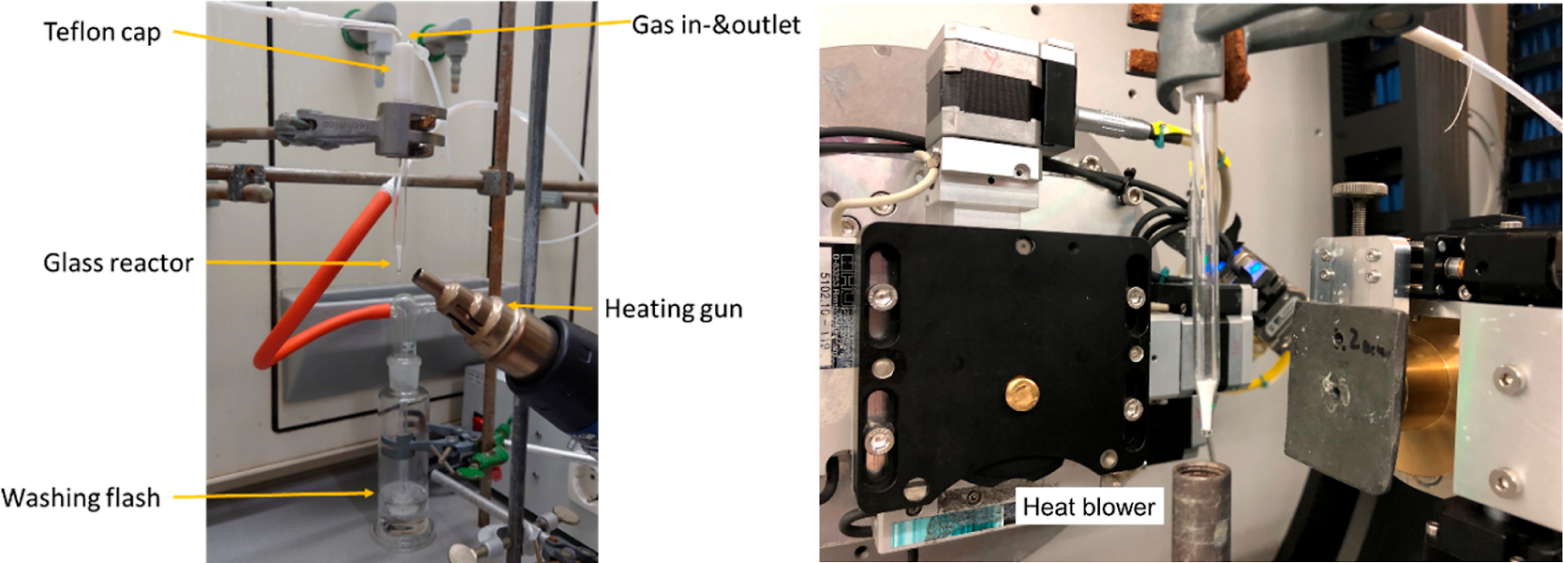
Setup designed for the synthesis experiments carried out in the laboratory (left). The same setup was used for the in situ XRPD experiments performed at DESY P02.1 (right).

The educts were filled into a specially designed glass reactor (Figure 1). The conical shape of the glass reactor provides an optimal scattering geometry for the XRPD experiments at the tip, while the wider part facilitates the release of exhaust gases. The shape also counteracts capillary forces and prevents the reaction mixture from moving out of the X-ray beam. The glass reactor was sealed with a Teflon cap equipped with a gas inlet and outlet system by which the reactor was maintained under a constant flow of argon (20 mL min^–1^). In case highly toxic phosphane gas would have formed during the reaction, the exhaust gas was passed through a hydrogen peroxide solution (36 wt %) for oxidizing phosphane to phosphates.

The reaction educts TiO_2_ (P25, Degussa, phase mixture of anatase and rutile, ≥99.5%) and NH_4_H_2_PO_2_ (Fluka, ≥97.0%) were mixed in various mass ratios of 1:4, 1:6, and 1:8. For a typical experiment, 30 mg of the reaction mixture was used.

In total, three in situ synchrotron XRPD experiments were conducted with different thermal treatments. The experimental parameters are summarized in Table 1.

**Table 1:** Overview of the Experiment Codes, Mass Ratios of the Educts, and Thermal Treatment Applied During the In Situ XRPD Experiments

no	experiment code	mass ratio TiO_2_/NH_4_H_2_PO_2_	thermal treatment heating rate/temperature/holding time/cooling rate
1	TiP-1^a^	1:6	20 °C min^–1^/320 °C/30 min/30 °C min^–1^
2	TiP-2^a^	1:4	20 °C min^–1^/320 °C/30 min/30 °C min^–1^
3	TiP-3^a^	1:8	20 °C min^–1^/320 °C/30 min/30 °C min^–1^
4	TiP-4^b^	1:6	20 °C min^–1^/275 °C/2 min/quenching
5	TiP-4R^b^	1:6	20 °C min^–1^/275 °C/2 min/quenching, reproduction experiment of TiP-4

a *In situ* experiments.

b Ex situ experiments.

### Ex Situ Synthesis

In addition to the in situ experiments, two ex situ samples were prepared in the laboratory (Table 1). A mixture of TiO_2_ and NH_4_H_2_PO_2_ (mass ratio 1:6) was heated at 20 °C min^–1^ to 275 °C under an argon flow and kept at this temperature for 2 min. The resulting powder samples TiP-4 and TiP-4R were quenched and washed to neutrality. A more detailed description of the synthesis procedure can be found in ref (27).

### Ion Exchange

**H–Ti(III)po** was prepared by a simple ion-exchange procedure by immersing **Ti(III)po** in an aqueous solution of dilute phosphoric acid. The assignment of the acronyms is explained in Table 2. In a standard experiment, 100 mg of **Ti(III)po** powder was immersed three times for 0.5 h in a 1 M aqueous solution (1 mL) of phosphoric acid in an ultrasonic bath at 40 °C. Afterward, the powder was washed three times with deionized water and dried overnight at 80 °C.

**Table 2:** Overview of the Structure Assignments Used in This Work, Chemical Formulas, and Space Groups

structure assignment	chemical formula	space group	reference
**Ti(III)p**	NH_4_TiP_2_O_7_	*P*2_1_/*c*	(28)
**Ti(III)po**	(NH4)_*x*_H_1–*x*_Ti(HPO_4_)_2_	*I*4_1_/*a*	this work
**H–Ti(III)po**	HTi(HPO_4_)_2_	*I*4_1_/*a*	this work
**Ti(IV)p**	TiP_2_O_7_	*P* 1̅	(28)

### Raman Spectroscopy

Spectra were recorded with an InVia Raman spectrometer (Renishaw Ltd., Wotton-under-Edge, UK). The sample was excited with a wavelength of 785 nm, and the laser power was reduced to 15 mW. The instrument was equipped with a 1200 mm^–1^ grating grid to provide a spectral resolution of 1 cm^–1^. The spectrum was collected with an exposure time of 10 s per step and three repetitions.

## Results and Discussion

### In Situ XRPD Experiments

**Figure 2:**
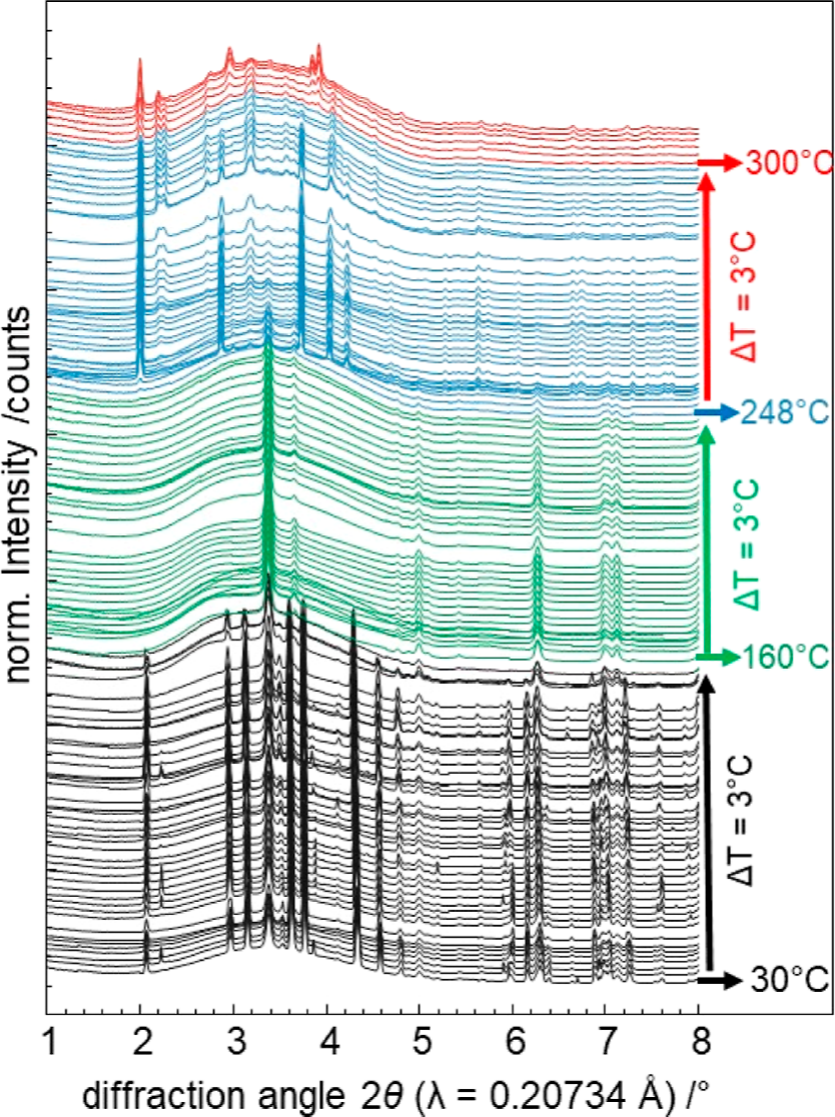
In situ temperature-dependent (TD) synchrotron XRPD data from the heating sequence of the experiment TiP-1 (Table 1, TiO_2_/NH_4_H_2_PO_2_ 1:6). Data were collected between 30 and 320 °C; the temperature increment between the individual data is 3 °C. The XRPD data are color-coded according to the observed phases: the educt mixture (NH_4_HPO_2_ and TiO_2_) in black, TiO_2_ in green, an intermediate phase **Ti(III)po** in blue, and **Ti(III)p** in red.

Figure 2 shows the in situ XRPD data recorded during the heating procedure applied for experiment TiP-1 (30–320 °C with a heating rate of 20 °C min^–1^). The starting compounds TiO_2_ and NH_4_H_2_PO_2_ (mass ratio 1:6) are detected from ambient temperature up to 160 °C (Figure 2, black data). NH_4_H_2_PO_2_ is highly crystalline with large crystallites, resulting in single-crystal reflections on the area detector. Above 130 °C, the crystallinity of NH_4_H_2_PO_2_ decreases, and typical powder rings are observed. Finally, NH_4_H_2_PO_2_ melts at 160 °C, and a mixture of white TiO_2_ in molten NH_4_H_2_PO_2_ is obtained. In the temperature range from 160 to 246 °C, only reflections of both TiO_2_ polymorphs (rutile and anatase) are detected (Figure 2, green data). The intensities of TiO_2_ decreases significantly above 200 °C, indicating either that the TiO_2_ crystallites become smaller or a reaction that consumes TiO_2_ is taking place. Simultaneously, a color change of the sample from white to deep blue is observed, indicating a reduction of Ti^4+^ to Ti^3+^. At 248 °C, a previously unknown highly crystalline intermediate phase (referred to as **Ti(III)po** in the following) is observed (Figure 2, blue data). At approximately 300 °C, the formation of **Ti(III)p** is detected (Figure 2, red data). Table 2 provides a summary of all structure names and chemical formulas.

A change of the mass ratio TiO_2_ to NH_4_H_2_PO_2_ from 1:6 (TiP-1) to 1:8 (TiP-3) does not affect the reaction pathway or the observed temperature regimes (Figure S1). However, reducing the NH_4_H_2_PO_2_ concentration (TiO_2_/NH_4_H_2_PO_2_ = 1:4) slows down the reaction to the product **Ti(III)p** (TiP-2; Figure S2). Here, **Ti(III)p** is observed only after a holding time of ∼2 min at 320 °C (Figure S3).

After dynamic heating to 320 °C, a holding sequence of 30 min at 320 °C was introduced. The XRPD data collected in experiments TiP-1 and TiP-3 show that **Ti(III)p** is the only crystalline phase at 320 °C (Figures S4 and S5). The time-dependent evolution of the unit cell parameters of the **Ti(III)p** structures shows a comparable slope (Figure S6). During the first 5 min, significant changes in the lattice parameters are observed. The cell volume follows this trend and decreases strongly in the first 5 min. All unit cell parameters decrease, except the *b* lattice parameter. In **Ti(III)p**, the TiO_6_ octahedra are interconnected via five P_2_O_7_^4–^ groups, four as a monodentate ligand and one as a bidentate ligand. In the *b-*direction, the neighboring TiO_6_ octahedra are connected via the bidentate P_2_O_7_^4–^ ligand, resulting in a rigid arrangement in this direction. As a consequence, the *b* lattice parameter increases during the first 5 min at 320 °C, while the other unit cell parameters decrease during structural relaxation after the formation of **Ti(III)p**. After 5 min, the metric parameters are stable.

As mentioned above, the conditions applied for the TiP-2 experiment slow the formation of **Ti(III)p** where the structural relaxation process takes about 15 min. This is also evident from the evaluated metric parameters, especially the *c* lattice parameter. In addition, the evaluation of the TiP-2 experiment shows a higher amount of impurities, identified as TiO_2_ and polyphosphates with reflections in the angular range between 3.0 and 3.7° 2θ. The deceleration of **Ti(III)p** formation is likely caused by an insufficient amount of the NH_4_H_2_PO_2_ melt. The latter facilitates the reaction and allows for unrestricted diffusion.

### Structure Determination of Ti(III)po

**Ti(III)po** crystallizes in the space group *I*4_1_/*a* [*a* = 6.3682(3) Å and *c* = 16.511(1) Å]. The obtained XRPD data of **Ti(III)po** are very similar to those of the literature-known Ti(HPO_4_)_2_ (Figure S7).^16^ However, as mentioned above, the deep blue color of the sample is characteristic of trivalent titanium cations (Ti^3+^). Figure S7 also shows the powder pattern of the reproduced synthesis product TiP-4R. To gain further information about the local coordination, the sample TiP-4 (Table 1) was analyzed by ex situ PDF analysis as well as by Raman and infrared (IR) spectroscopies.

**Figure 3:**
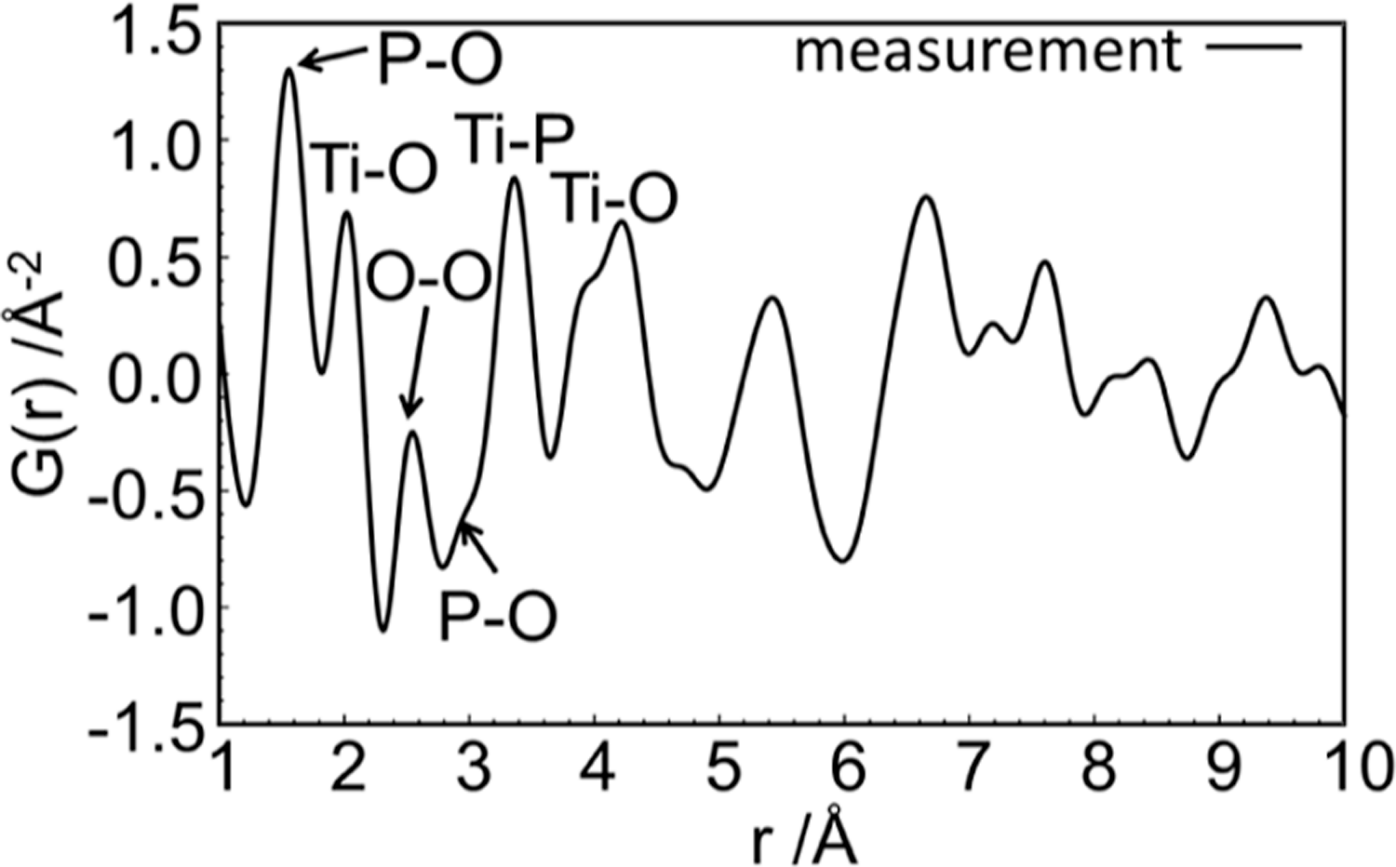
Ex situ PDF analysis of the intermediate phase **Ti(III)po**.

In the qualitative ex situ PDF analysis (Figure 3), atom pair correlations at 1.562 and 2.536 Å are observed fitting well to known P–O (1.50–1.58 Å) and O–O (2.45–2.55 Å) distances of phosphate compounds.^21,22^ The Ti–O pair correlation at 2.021 Å is significantly elongated compared to typical Ti^4+^–O bond distances, varying between 1.885(1) and 1.945(1) Å.^16,32^ However, the correlation is in good agreement with Ti^3+^–O bond distances between 2.033(2) and 2.029(2) Å as determined for Ti(PO_3_)_3_.^33^ This provides further evidence that **Ti(III)po** contains Ti^3+^ in the bulk structure.

For condensed phosphates, P–P correlations of 2.84–2.98 Å are expected. As such, for pyrophosphates, P–P correlations at 2.99 Å [TiP_2_O_7_ (*Pa*3̅)] and PO_4_ chains or rings at 2.87–2.97 Å [FeP_3_O_9_ (*Cc*) P–P = 2.95–2.99 Å and K_3_P_3_O_9_ (*P*2_1_/*n*1) P–P = 2.89–2.93 Å] are expected.^32,34,35^ As the PDF data do not show such P–P correlations for **Ti(III)po**, it is concluded that the **Ti(III)po** structure belongs to the group of orthophosphates.

**Figure 4:**
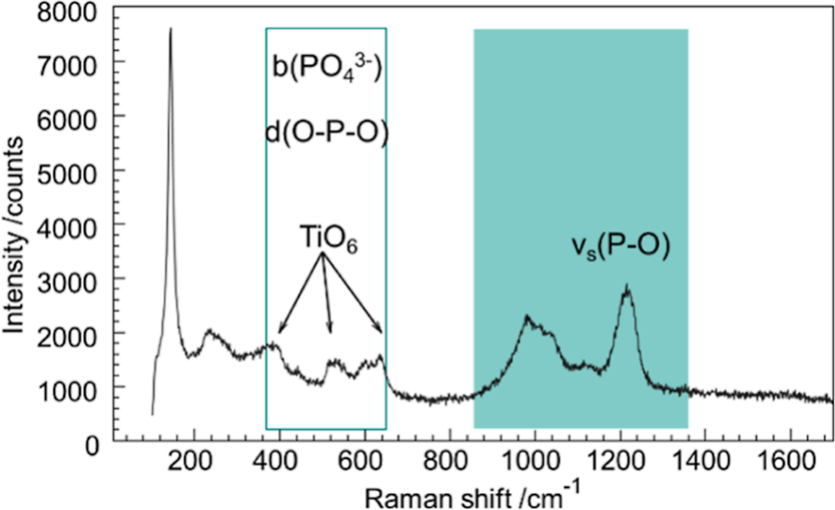
Raman spectra (785 nm) of **Ti(III)po**; the characteristic modes of TiO_6_ and PO_*x*_ are marked.

The local coordination of **Ti(III)po** was further analyzed via Raman spectroscopy (Figure 4). The symmetric stretching vibrations of the PO_*x*_ polyhedra [ν_s_(P–O)] are found in the range of ∼900–1200 cm^–1^.^32,36,37^ The Raman shifts of these modes are highly sensitive to the bond distances, which are affected by the secondary coordination shell of the PO_*x*_ polyhedra.^32^ This correlation allows the assignment of orthophosphates [ν_s_(P–O) = 900–1100 cm^–1^ and *r*(P–O) = 1.50–1.58 Å], pyrophosphates [ν_s_(P–O) = 975–1250 cm^–1^ and *r*(P–O) = 1.45–1.56 Å], and metaphosphates [ν_s_(P–O) = 1050–1150 cm^–1^ and *r*(P–O) = 1.45–1.54 Å].^32^ The symmetric stretching vibrations ν_s_(P–O) of **Ti(III)po** are observed in the spectral range from 980 to 1215 cm^–1^. These Raman shifts correlate well with the orthophosphate and pyrophosphate groups. However, neither the characteristic symmetric stretching [ν_s_(P–O–P) = 750 cm^–1^] nor the deformation vibrations [δ(P–O–P) = 910 cm^–1^] for pyrophosphate units are detected.^32,36^ In the range of ∼370–650 cm^–1^, PO_4_^3–^ bending b(PO_4_^3–^) and deformation *d*(O–P–O) vibrations are observed. The absence of ν_s_(P–O–P) and δ(P–O–P) and the Raman shift of ν_s_(P–O) indicate that **Ti(III)po** consists of orthophosphates, which is in good agreement with the results of the PDF analysis. The characteristic vibrations of TiO_6_ octahedra (399, 519, and 639 cm^–1^) are also detected.^36−38^

**Figure 5:**
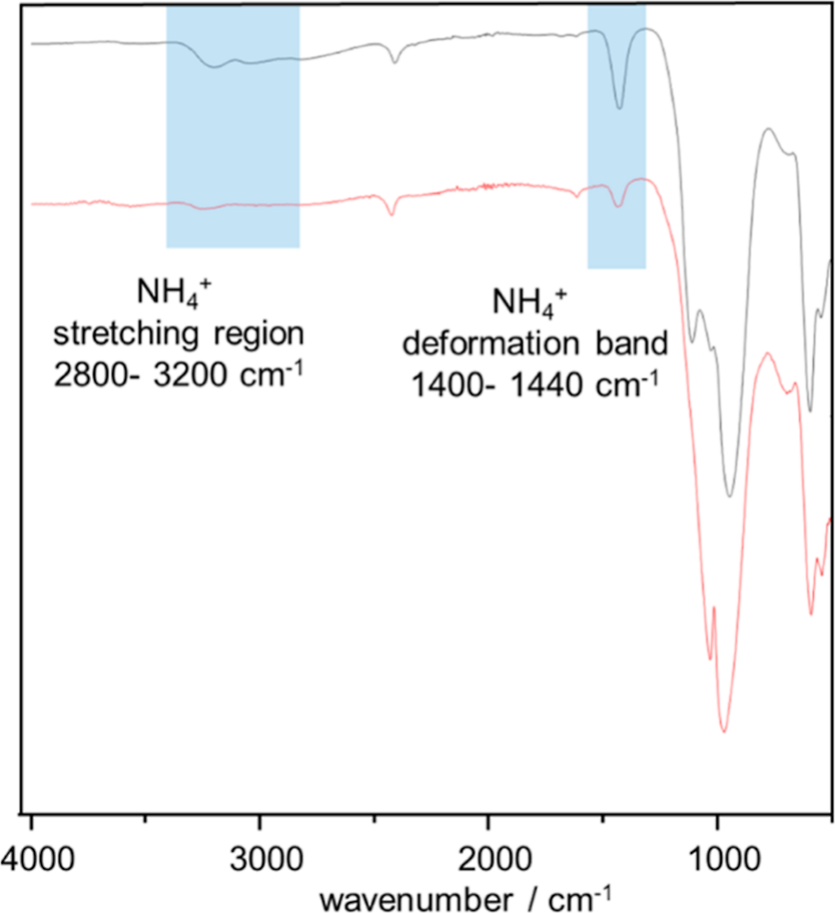
IR spectra of **Ti(III)po** (black curve) and the proton-exchanged sample **H–Ti(III)po** (red curve). In blue, the characteristic regions of NH_4_^+^ modes are marked.

In the IR spectrum of **Ti(III)po** (Figure 5), the characteristic stretching (2800–3200 cm^–1^) and deformation modes (1400–1440 cm^–1^) of NH_4_^+^ are visible, suggesting the incorporation of NH_4_^+^ cations into the structure.^38^ Below 1300 cm^–1^, typical vibrations of phosphate units are observed. The vibrations at ∼2300 cm^–1^ and 1120–1200 cm^–1^ can be correlated with symmetric stretching vibrations of P–H and asymmetric stretching vibrations of PO_4_ units. However, these vibrations are considered to originate from the amorphous phosphate glasses produced during the synthesis process.^9,39^

**Figure 6:**
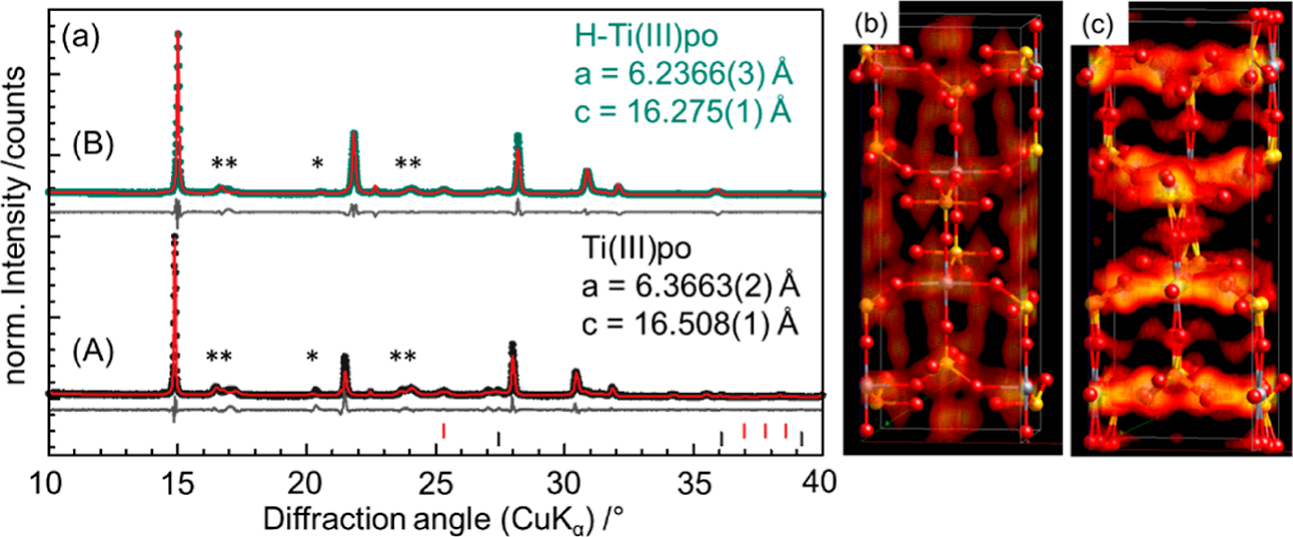
(a) Rietveld refinement plots of **Ti(III)po** (A, black) and **H–Ti(III)po** (B, green). The measured data are displayed as points; the red line represents the calculated data from the respective model. The difference curve is shown in gray. In addition, the corresponding difference Fourier maps are shown: (b) **Ti(III)po** and (c) **H–Ti(III)po**. Polyphosphate is marked by asterisks.

**Figure 7:**
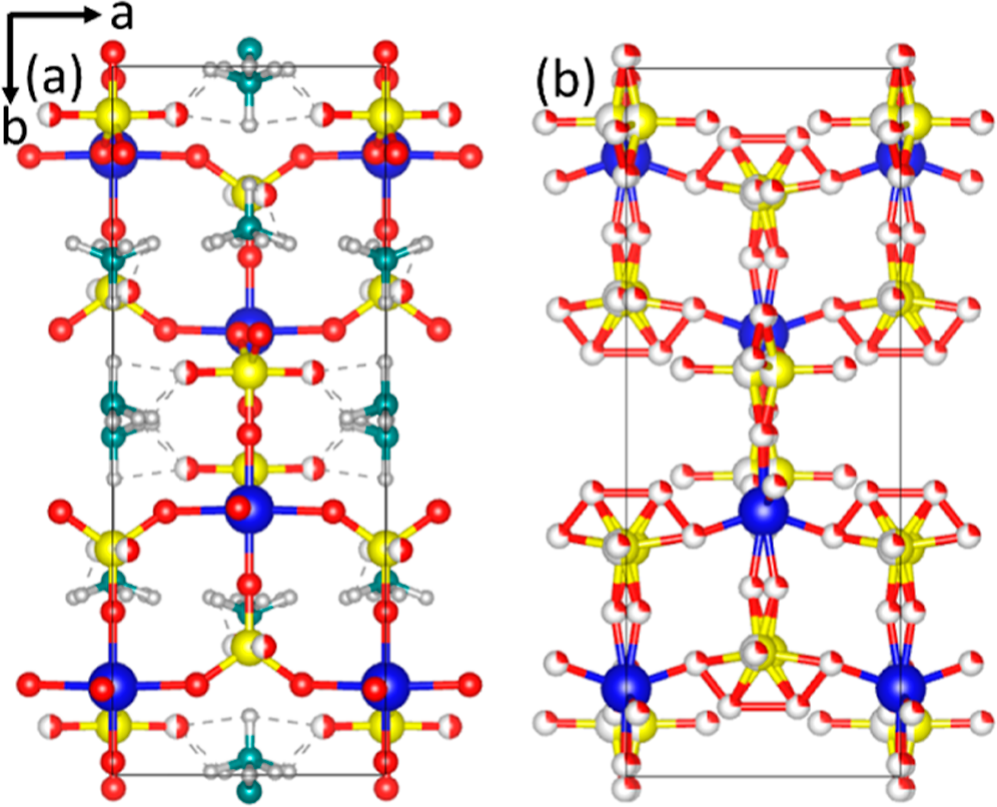
(a) Crystal structure of **Ti(III)po** together with the (b) proton-exchanged counterpart **H–Ti(III)po**. Color code: blue: Ti, yellow: P, green: N, red: O, gray: H, and red/white: split position H and O.

The XRPD data collected for TiP-4 (Figure 6) show reflections of **Ti(III)po** and impurities (polyphosphates and TiO_2_ in the rutile as well as the anatase modification). Figure S7 shows for comparison the XRPD data of TiP-4 and a reproduced sample TiP-4R. Both products were obtained using the conditions described in Table 1 (TiP-4). In both synthesis products, **Ti(III)po** is the major phase, whereas the fraction of the ammonium polyphosphate (*C*222_1_) impurity (marked with asterisks) varies. For the structure determination of **Ti(III)po**, the reflections were indexed (space group *I*4_1_/*a*), and the metric parameters [*a* = 6.3682(3) Å and *c* = 16.511(1) Å] were obtained by subsequent Pawley fitting. Furthermore, orthophosphate units (PO_4_^3–^) were introduced as rigid bodies, and titanium was considered in the trivalent state (Ti^3+^). The resulting structure, determined by simulated annealing, closely resembles the structure of Ti(HPO_4_)_2_ (Figure S8). However, the IR data imply incorporation of NH_4_^+^ cations into the structure. The position of the NH_4_^+^ cations in the crystal structure was therefore analyzed using the charge-flipping algorithm implemented in DiffracPlus TopasV6 (Figure S9). Without considering NH_4_^+^ cations in the structure, a significant rest of the electron density was detected in the channel structure running along the crystallographic *c*-axis. This results in a central NH_4_^+^ position at Wyckoff position 8*e* (0, 0.25, and 0.02236) (Table S1). For **Ti(III)po**, the structure analysis thus results in a compound with the composition (NH_4_)_*x*_H_1–*x*_Ti(HPO_4_)_2_ with *x* = 0.41 (Figure 7).

The structure of **Ti(III)po** consists of Ti^3+^O_6_ octahedra coordinated by six orthophosphate units H_*n*_(HPO_4_)^(2–*n*)–^ and NH_4_^+^ cations. The local arrangement results in the formation of cavities along the crystallographic *c*-axis. The hydroxyl groups as well as the NH_4_^+^ cations are either directed toward or located in these cavities. The TiO_6_ octahedron consists of a fairly regular plane with a Ti–O distance of 1.970(1) Å and a shortened axial distance of 1.752(1) Å. The observed O1–Ti–O2 angles are close to 90° and range from 88.60(2)° to 91.40(2)° (Table S2). The obtained distances and angles are in good agreement with reported values.^28,33^

### Ion-Exchange Experiments

A comparison of the XRPD data collected for **Ti(III)po** before and after the exchange of NH_4_^+^ by H^+^ is displayed in Figure 6. The protonated sample is denoted as **H–Ti(III)po**. A significant shift in the reflection position of **H–Ti(III)po** to higher angles is observed. The shift corresponds to a decrease in the lattice parameters *a* = 6.3266(3) Å [Ti(III)po: *a* = 6.3663(2) Å) and *c* = 16.275(1) Å (Ti(III)po: *c* = 16.508(1) Å], indicating a successful exchange of the larger NH_4_^+^ ions by H^+^. In addition, the NH_4_^+^ modes in the IR spectrum of **Ti(III)po** become less intense after exchange (Figure 5). However, the exchange of NH_4_^+^ by H^+^ is apparently not complete. A small signal indicates that a small proportion of NH_4_^+^ ions remains in the **H–Ti(III)po** structure. That minor fraction of NH_4_^+^ is not considered in the structure analysis though. For the crystal structure refinement of **H–Ti(III)po**, a rigid body was used for the orthophosphate units assuming P–O distances between 1.46 and 1.65 Å and an O–P– O angle of 109.28°. The structure of the proton-exchanged sample consists of TiO_6_ octahedra with trivalent titanium coordinated by six orthophosphate units (HPO_4_)^2–^, similar to that of **Ti(III)po** (Figure 7). The overall network of local coordination polyhedra remains stable during the exchange. However, the orthophosphate units are shifted from special position 8*e* (0.5, 0.25, *z*) to 16*f* (*x*, *y*, *z*), showing a local disorder due to the exchange of NH_4_^+^ by H^+^ (Table S3). The P–O distance was determined to be 1.562(5) Å. In the TiO_6_ octahedra, the Ti–O distances range from 1.75(9) to 1.78(1) Å, while the O–Ti–O angles show values between 62(3)° and 167(4)° (Table S4). In general, the crystal structure of **H– Ti(III)po** shows a higher degree of disorder in the local coordination environment. Comparing the difference Fourier map of **H–Ti(III)po** with the one obtained for **Ti(III)po** without considering the NH_4_^+^ cations shows significant differences in the residual electron density at the potential NH_4_^+^ position, proving indirect evidence for the presence of NH_4_^+^ at this position (Figure 6).

**Figure 8:**
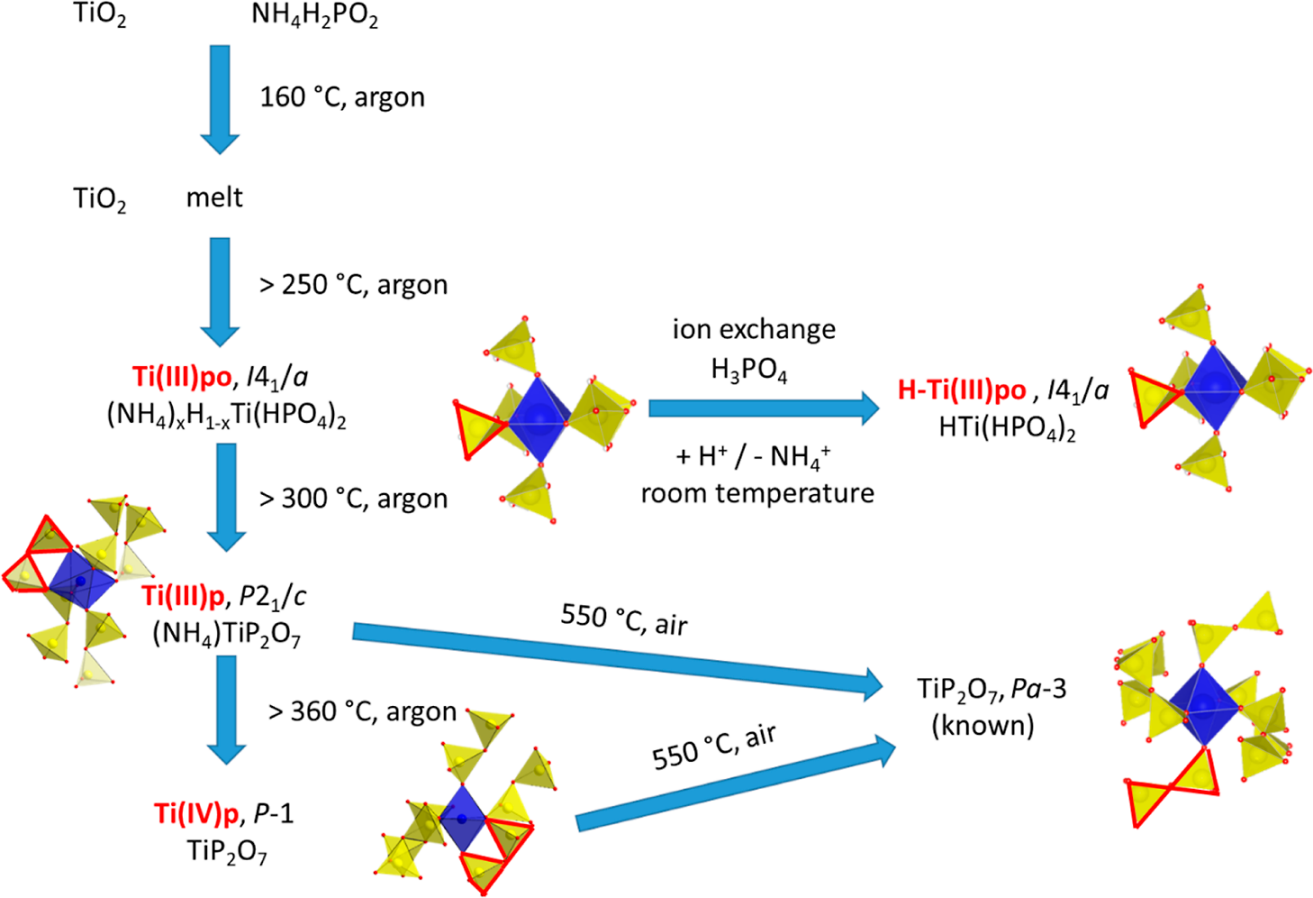
Reaction pathway and relation of known and novel titanium phosphate phases.

Figure 8 summarizes the temperature-driven reaction pathway starting from TiO_2_ and NH_4_H_2_PO_2_, highlighting the formation of different intermediates and final structures along with the crystal structures resolved in this study or reported in previous work.

## Conclusions

The study of the reductive phosphatization by in situ temperature-dependent synchrotron XRPD experiments on NH_4_H_2_PO_2_ and TiO_2_ reveals a multistep reaction to NH_4_TiP_2_O_7_, **Ti(III)p** (Figure 8). At about 160 °C, NH_4_H_2_PO_2_ melts, and only TiO_2_ reflections are observed. The intensities of the reflections start to decrease at higher temperatures. At the same time, a color change at approximately 200 °C from white to deep blue indicates the reduction of Ti^4+^ to Ti^3+^ and simultaneous oxidation of the hypophosphite precursor. At 248 °C, a new highly crystalline intermediate phase (**Ti(III)po**) with the chemical composition (NH_4_)_*x*_H_1–*x*_(HPO_4_)_2_ with *x* = 0.41 is observed. **Ti(III)po** crystallizes in the space group *I*4_1_/*a* and consists of regular TiO_6_ octahedra with Ti^3+^ ions. The TiO_6_ octahedra are connected via six orthophosphate units (HPO_4_)^2–^. The local arrangement results in the formation of cavities along the crystallographic *c-*axis. The hydroxyl groups of (HPO_4_)^2–^ are directed toward and the NH_4_^+^ cations are located in these cavities. The formation of monoclinic **Ti(III)p** NH_4_TiP_2_O_7_, space group *P*2_1_/*c*, proceeds at about 300 °C and requires the polycondensation of the (HPO_4_)^2–^ units to pyrophosphate units. The new compound shows good proton exchangeability. Through proton exchange, the NH_4_^+^ position could be indirectly proven. The evolution of the unit cell parameters of **Ti(III)p** at high temperatures over time shows a subsequent structural relaxation.

The variation of the educt ratios (TiO_2_/NH_4_H_2_PO_2_ = 1:4, 1:6, and 1:8) shows that a weight ratio of at least TiO_2_/NH_4_H_2_PO_2_ = 1:6 is required for a quantitative and fast reaction of TiO_2_ and NH_4_H_2_PO_2_ to **Ti(III)po** with a low amount of impurities. The experiments indicate that NH_4_H_2_PO_2_ acts not only as a reductive precursor but also as the reaction medium for the reaction, in which the crystallization proceeds during the heating process. Reducing the concentration hinders the diffusion of the reactants and decelerates the reaction. The complete reaction proceeds in five successive steps, i.e., melting of NH_4_H_2_PO_2_, a first redox reaction, the formation of tetragonal **Ti(III)po** (NH_4_)_*x*_H_1–*x*_Ti(HPO_4_)_2_, the formation of monoclinic **Ti(III)p**, and finally a structural relaxation of **Ti(III)p**. The intermediate **Ti(III)po** could not be obtained by previous ex situ experiments, which demonstrates the importance of in situ XRD studies as performed here. Only such studies allow for the identification of all phases present under certain reaction conditions. Here, we can clearly show that different phosphate phases crystallize successively within the ammonium hypophosphite melt and remain stable during the cooling process.

## Supporting Information Available

In situ temperature-dependent synchrotron XRPD patterns, Rietveld refinement plot, crystal structure plots of Ti(IV)(HPO_4_)_2_ and Ti(III)po, difference Fourier analysis plots of Ti(III)po, crystal structure data, and interatomic bonds and angles of Ti(III)po and H–Ti(III)po (PDF)

## Abbreviations

TMPs: transition metal phosphates,
TiO_2_: titania,
NH_4_H_2_PO_2_: ammonium hypophosphite,
XRPD: X-ray powder diffraction,
[PO_4_]^3–^: orthophosphates,
[P_2_O_7_]^4–^: pyrophosphates,
[P_*n*_O3_*n*_]^*n*–^: metaphosphates,
PDF: pair distribution function
IR: infrared

## References

[ref1] Deniard P.; Dulac A. M.; Rocquefelte X.; Grigorova V.; Lebacq O.; Pasturel A.; Jobic S. High potential positive materials for lithium-ion batteries: transition metal phosphates. J. Phys. Chem. Solids 2004, 65 (2), 229–233. 10.1016/j.jpcs.2003.10.019.

[ref2] Jin Y.; Shen Y.; Hibino T. Proton conduction in metal pyrophosphates (MP_2_O_7_) at intermediate temperatures. J. Mater. Chem. 2010, 20, 6214–6217. 10.1039/b924188d.

[ref3] Lin R.; Ding Y. A. Review on the Synthesis and Applications of Mesostructured Transition Metal Phosphates. Mater. Res. Bull. 2013, 6, 217–243. 10.3390/ma6010217. PMC5452118 28809304

[ref4] Yang S.; Zavalij P. Y.; Stanley Whittingham M. Hydrothermal synthesis of lithium iron phosphate cathodes. Electrochem. Commun. 2001, 3 (9), 505–508. 10.1016/S1388-2481(01)00200-4.

[ref5] Clark J. M.; Nishimura S. I.; Yamada A.; Islam M. S. Insights into Local Structure and Lithium-Diffusion Pathways. Angew. Chem., Int. Ed. 2012, 51 (52), 13149–13153. 10.1002/anie.201205997. 23154889

[ref6] Hong L.; Li L.; Chen-Wiegart Y. K.; Wang J.; Xiang K.; Gan L.; Li W.; Meng F.; Wang F.; Wang J.; Chiang Y. M.; Jin S.; Tang M. Two-dimensional lithium diffusion behavior and probable hybrid phase transformation kinetics in olivine lithium iron phosphate. Nat. Commun. 2017, 8 (1), 1194 10.1038/s41467-017-01315-8. 29084965 PMC5662729

[ref7] Lee S.; Park S. S. Structure, Defect Chemistry, and Lithium Transport Pathway of Lithium Transition Metal Pyrophosphates (Li_2_MP_2_O_7_, M: Mn, Fe, and Co): Atomistic Simulation Study. Chem. Mater. 2012, 24 (18), 3550–3557. 10.1021/cm301921d.

[ref8] Jugović D.; Uskoković D. A review of recent developments in the synthesis procedures of lithium iron phosphate powders. J. Power Sources 2009, 190 (2), 538–544. 10.1016/j.jpowsour.2009.01.074.

[ref9] Zhang W. J. Structure and performance of LiFePO_4_ cathode materials: A review. J. Power Sources 2011, 196 (6), 2962–2970. 10.1016/j.jpowsour.2010.11.113.

[ref10] Li H.; Xing S.; Liu Y.; Li F.; Guo H.; Kuang G. Recovery of Lithium, Iron, and Phosphorus from Spent LiFePO4 Batteries Using Stoichiometric Sulfuric Acid Leaching System. ACS Sustain. Chem. Eng. 2017, 5 (9), 8017–8024. 10.1021/acssuschemeng.7b01594.

[ref11] Hutchings G. J. Vanadium phosphate: a new look at the active components of catalysts for the oxidation of butane to maleic anhydride. J. Mater. Chem. 2004, 14, 3385–3395. 10.1039/b404610m.

[ref12] Hutchings G. J.; Kiely C. J.; Sananes-Schulz M. T.; Burrows A.; Volta J. C. Comments on the nature of the active site of vanadium phosphate catalysts for butane oxidation. Catal. Today 1998, 40 (2), 273–286. 10.1016/S0920-5861(98)00015-7.

[ref13] Contractor R. M.; Bergna H. E.; Horowitz H. S.; Blackstone C. M.; Malone B.; Torardi C. C.; Griffiths B.; Chowdhry U.; Sleight A. W. Butane oxidation to maleic anhydride over vanadium phosphate catalysts. Catal. Today 1987, 1 (1), 49–58. 10.1016/0920-5861(87)80026-3.

[ref14] Coulston G. W.; Bare S. R.; Kung H.; Birkeland K.; Bethke G. K.; Harlow R.; Herron N.; Lee P. L. The Kinetic Significance of V^5+^ in n-Butane Oxidation Catalyzed by Vanadium Phosphates. Science 1997, 275 (5297), 191 10.1126/science.275.5297.191. 8985008

[ref15] Stegmann N.; Ochoa-Hernández C.; Truong K. N.; Petersen H.; Weidenthaler C.; Schmidt W. The Mechanism and Pathway of Selective Partial Oxidation of n-Butane to Maleic Anhydride Studied on Titanium Phosphate Catalysts. ACS Catal. 2023, 13 (24), 15833–15840. 10.1021/acscatal.3c03172.

[ref16] Ekambaram S.; Serre C.; Férey G.; Sevov S. C. Hydrothermal Synthesis and Characterization of an Ethylenediamine-Templated Mixed-Valence Titanium Phosphate. Chem. Mater. 2000, 12 (2), 444–449. 10.1021/cm990492i.

[ref17] Nalini V.; Sørby M. H.; Amezawa K.; Haugsrud R.; Fjellvåg H.; Norby T. Structure, Water Uptake, and Electrical Conductivity of TiP_2_O_7_. J. Am. Ceram. Soc. 2011, 94 (5), 1514–1522. 10.1111/j.1551-2916.2010.04281.x.

[ref18] Levi G. R.; Peyronel G.; Peyronel G. Struttura Cristallografica del Gruppo Isomorfo (Si^4+^, Ti^4+^, Zr^4+^, Sn^4+^, Hf^4+^)P_2_O_7_. Z. Kristallogr. 1935, 92, 190–209. 10.1524/zkri.1935.92.1.190.

[ref19] Sanz J.; Iglesias J. E.; Soria J.; Losilla E. R.; Aranda M. A. G.; Bruque S. Structural Disorder in the Cubic 3 × 3 × 3 Superstructure of TiP_2_O_7_. XRD and NMR Study. Chem. Mater. 1997, 9 (4), 996–1003. 10.1021/cm970057t.

[ref20] Norberg S. T.; Svensson G.; Abertsson J. A. TiP_2_O_7_ superstructure. Acta Crystallogr. C 2001, C57, 225–227. 10.1107/s0108270100018709 11250555

[ref21] Septiani N. L. W.; Kaneti Y. V.; Fathoni K. B.; Kani K.; Allah A. E.; Yuliarto B.; Yamauchim Y.; Dipojono H. K.; Alothman Z. A.; Golberg D.; et al. Self-Assembly of Two-Dimensional Bimetallic Nickel–Cobalt Phosphate Nanoplates into One-Dimensional Porous Chainlike Architecture for Efficient Oxygen Evolution Reaction. Chem. Mater. 2020, 32, 7005–7018. 10.1021/acs.chemmater.0c02385.

[ref22] Lin R.; Ding Y. A Review on the Synthesis and Applications of Mesostructured Transition Metal Phosphates. Materials 2013, 6, 217–243. 10.3390/ma6010217. 28809304 PMC5452118

[ref23] Kuo D. H.; Tseng W. C. Amorphous Ti-P-O films grown with four-component chemical vapor deposition. Mater. Chem. Phys. 2005, 93, 361–3367. 10.1016/j.matchemphys.2005.03.023.

[ref24] Holdsworth A. F.; Eccles H.; Halman A. M.; Mao R.; Bond G. Low-Temperature Continuous Flow Synthesis of Metal Ammonium Phosphates. Sci. Rep. 2018, 8, 13547 10.1038/s41598-018-31694-x. 30201951 PMC6131346

[ref25] Dobbelaere T.; Mattelaer F.; Dendooven J.; Vereecken P.; Detavernier C. Plasma-Enhanced Atomic Layer Deposition of Iron Phosphate as a Positive Electrode for 3D Lithium-Ion Microbatteries. Chem. Mater. 2016, 28, 3435–3445. 10.1021/acs.chemmater.6b00853.

[ref26] Syed N.; Zavabeti A.; Ou J. Z.; Mohiuddin M.; Pillai N.; Carey B. J.; Zhang B. Y.; Datta R. S.; Jannat A.; Haque F.; Messalea K. A.; Xu C.; Russo S. P.; McConville C. F.; Daeneke T.; Kalantar-Zadeh K. Printing two-dimensional gallium phosphate out of liquid metal. Nat. Commun. 2018, 9, 3618 10.1038/s41467-018-06124-1. 30190463 PMC6127148

[ref27] Stegmann N.; Petersen H.; Weidenthaler C.; Schmidt W. Facile synthesis of novel, known, and low-valent transition metal phosphates via reductive phosphatization. J. Mater. Chem. A 2021, 9, 18247–18250. 10.1039/D1TA03782J.

[ref28] Petersen H.; Stegmann N.; Fischer M.; Zibrowius B.; Radev I.; Philippi W.; Schmidt W.; Weidenthaler C. Crystal Structures of Two Titanium Phosphate-Based Proton Conductors: Ab Initio Structure Solution and Materials Properties. Inorg. Chem. 2022, 61 (5), 2379–2390. 10.1021/acs.inorgchem.1c02613. 34807595 PMC8826274

[ref29] Coelho A. A. *TOPAS* and *TOPAS-Academic*: an optimization program integrating computer algebra and crystallographic objects written in C++. J. Appl. Crystallogr. 2018, 51 (1), 210–218. 10.1107/s1600576718000183.

[ref30] Juhas P.; Davis T.; Farrow C. L.; Billinge S. J. L. PDFgetX3: a rapid and highly automatable program for processing powder diffraction data into total scattering pair distribution functions. J. Appl. Crystallogr. 2013, 46 (2), 560–566. 10.1107/S0021889813005190.

[ref31] Filik J.; Ashton A. W.; Chang P. C. Y.; Chater P. A.; Day S. J.; Drakopoulos M.; Gerring M. W.; Hart M. L.; Magdysyuk O. V.; Michalik S.; Smith A.; Tang C. C.; Terrill N. J.; Wharmby M. T.; Wilhelm H. Processing two-dimensional X-ray diffraction and small-angle scattering data in DAWN 2. J. Appl. Crystallogr. 2017, 50, 959–966. 10.1107/S1600576717004708. 28656043 PMC5458597

[ref32] Popović L.; de Waal D.; Boeyens J. C. A. Correlation between Raman wavenumbers and P-O bond lengths in crystalline inorganic phosphates. J. Raman Spectrosc. 2005, 36 (1), 2–11. 10.1002/jrs.1253.

[ref33] Harrison W. T. A.; Gier T. E.; Stucky G. D. Titanium(III) tris(metaphosphate). Acta Crystallogr., Sect. C: Cryst. Struct. Commun. 1994, 50 (11), 1643–1646. 10.1107/S010827019400613X.

[ref34] Elbouaanani L. K.; Malaman B.; Gérardin R. Structure refinement and magnetic properties of C–Fe (PO_3_) _3_ studied by neutron diffraction and Mössbauer techniques. J. Solid State Chem. 1999, 148 (2), 455–463. 10.1006/jssc.1999.8479.

[ref35] Bagieu-Beucher M.; Tordjman I.; Durif A.; Guitel J. C. Structure cristalline du trimétaphosphate de potassium K_3_P_3_O_9_. Acta Crystallogr. B 1976, 32 (5), 1427–1430. 10.1107/S0567740876005505.

[ref36] Dardar F. E.; Gross M.; Krimi S.; Couzi M.; Lachgar A.; Sebti S.; El Jazouli A. Synthesis, structural characterization and ionic conductivity of mixed alkali titanium phosphate glasses. Mediterr. J. Chem. 2018, 7 (5), 328–336. 10.13171/mjc751912040810aej.

[ref37] Schmutz C.; Barboux P.; Ribot F.; Taulelle F.; Verdaguer M.; Fernandez-Lorenzo C. EXAFS, Raman and 31P NMR study of amorphous titanium phosphates. J. Non-Cryst. Solids 1994, 170, 250–262. 10.1016/0022-3093(94)90054-X.

[ref38] Nakamoto K. Infrared and Raman Spectra of Inorganic and Coordination Compounds Part A: Theory and Applications in Inorganic Chemistry; John Wiley & Sons, Inc., 2009.

[ref39] Wu J.; Dathar G. K. P.; Sun C.; Theivanayagam M. G.; Applestone D.; Dylla A. G.; Manthiram A.; Henkelman G.; Goodenough J. B.; Stevenson K. J. *In situ* Raman spectroscopy of LiFePO_4_: size and morphology dependence during charge and self-discharge. Nat. Nanotechnol. 2013, 24 (42), 424009 10.1088/0957-4484/24/42/424009. 24067625

