# Supplementary material for: Investigating the Reductive Phosphatization Reaction Pathway in the Synthesis of Transition Metal Phosphates: A Case Study on Titanium Phosphates
Source: Inorg Chem. 2025 Jan 24;64(5):2425–32. doi: 10.1021/acs.inorgchem.4c04776 (PMC11815834; doi:10.1021/acs.inorgchem.4c04776)
Supplement: Supplementary file 1 — ic4c04776_si_001.pdf [file ic4c04776_si_001.pdf]

Supporting Information

Investigating the Reductive Phosphatization Reaction Pathway in the Synthesis of Transition Metal Phosphates: A Case Study on Titanium Phosphates

*Hilke Petersen[1], Niklas Stegmann[1], Wolfgang Schmidt[1], and Claudia Weidenthaler[1]**

[1]Max-Planck-Institut für Kohlenforschung, Heterogeneous Catalysis, Kaiser-Wilhelm-Platz 1

45470 Mülheim, Germany

*Corresponding author: weidenthaler@mpi-muelheim.mpg.de

Table of Contents:

Figure S1. *In situ* XRPD data, experiment TiP-3……………………………………..S2

Figure S2: *In situ* XRPD data experiment TiP-2……………………………………...S3

Figure S3: *In situ* XRPD data, TiO_2 : $NH_4H_2PO_2$ 1 : 4……………………………….S4

Figure S4. *In situ* XRPD data, TiO_2 : $NH_4H_2PO_2$ 1 : 6……………………………….S5

Figure S5. *In situ* XRPD data, TiO_2 : $NH_4H_2PO_2$ 1 : 8……………………………….S6

Figure S6. Structure parameters **Ti(III)p**……………………………………………..S7

Figure S7. Rietveld plots **Ti(III)po**……………………………………………………..S8

Figure S8. Crystal structures of **Ti(IV)(HPO$_4$)$_2$** and **Ti(III)po**…………………………S9

Figure S9. Difference Fourier analysis of **Ti(III)po**……………………………………S10

Table S1. Crystal structure data of **Ti(III)po**…………………………………………...S11

Table S2. Interatomic distances and angles of **Ti(III)po**………………………………S12

Table S3: Crystal structure data of **H-Ti(III)po**………………………………………..S13

Table S4: Selected interatomic distances and angles of **H-Ti(III)po**…………………..S14

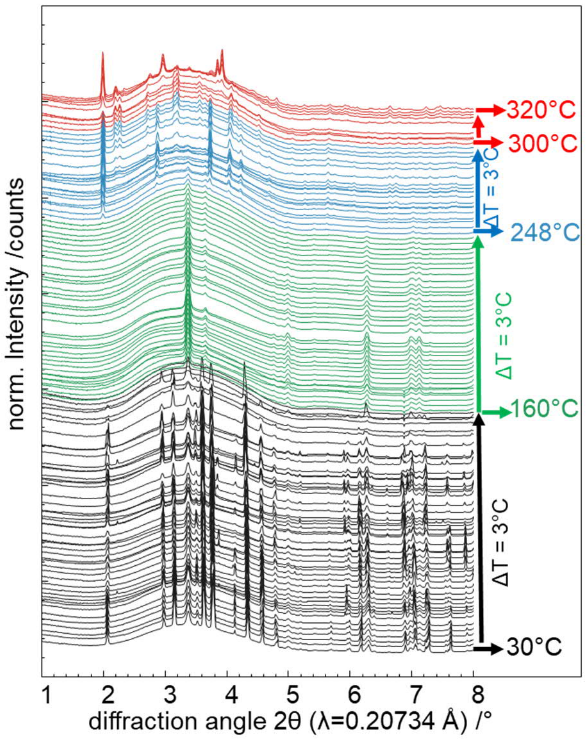

Figure S1. *In situ* temperature-dependent synchrotron XRPD data from the heating sequence of the experiment TiP-3 (compare Table 1; reaction mixture TiO_2 : $NH_4H_2PO_2$ 1 : 8). The XRPD data is color-coded with respect to the observed phases: the educt mixture ($NH_4H_2PO_2$ and TiO_2) in black, TiO_2 in green, intermediate **Ti(III)po** in blue, and **Ti(III)p** ($NH_4TiP_2O_7$) in red. Data were collected between 30 and 320 °C with a heating rate of 20 K min^{-1}. Data accumulation time was 60 s, data are plotted with an increment of 3 °C.

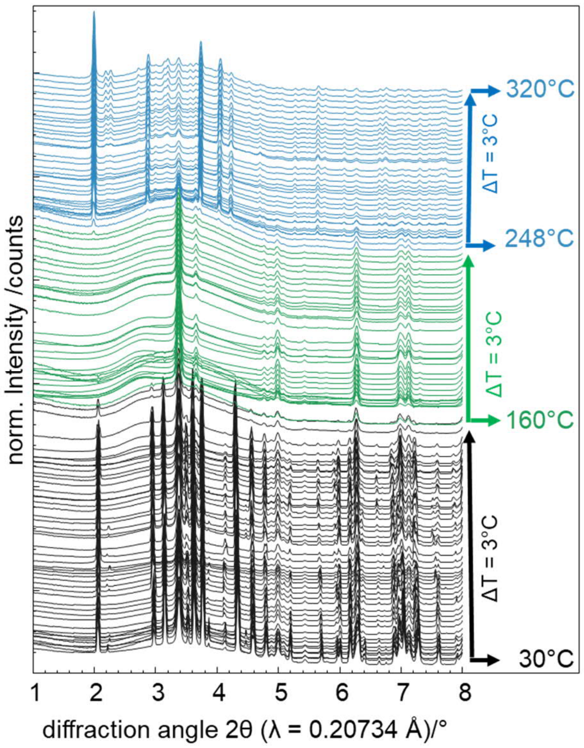

Figure S2. *In situ* temperature-dependent synchrotron XRPD data from the heating sequence of the experiment TiP-2 (Table 1; reaction mixture TiO_2 : $NH_4H_2PO_2$ 1 : 4). The XRPD data is color-coded with respect to the observed phases: the educt mixture ($NH_4H_2PO_2$ and TiO_2) in black, TiO_2 in green, **Ti(III)po** in blue. Data were collected between 30 and 320 °C with a heating rate of 20 K min^{-1}. Data accumulation time was 60 s, data are plotted with an increment of 3 °C.

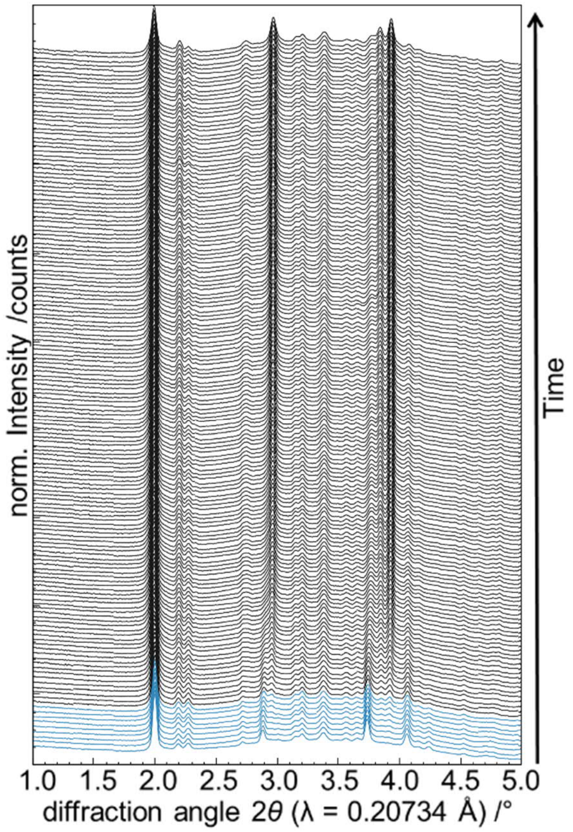

Figure S3. *In situ* time-dependent synchrotron XRPD data from the holding sequence of the experiment TiP-2 (compare Table 1; reaction mixture TiO_2 : $NH_4H_2PO_2$ 1 : 4) at 320 °C for 30 min. The XRPD data is color-coded with respect to the observed phases: the intermediate **Ti(III)po** in blue, and **Ti(III)p** in black.

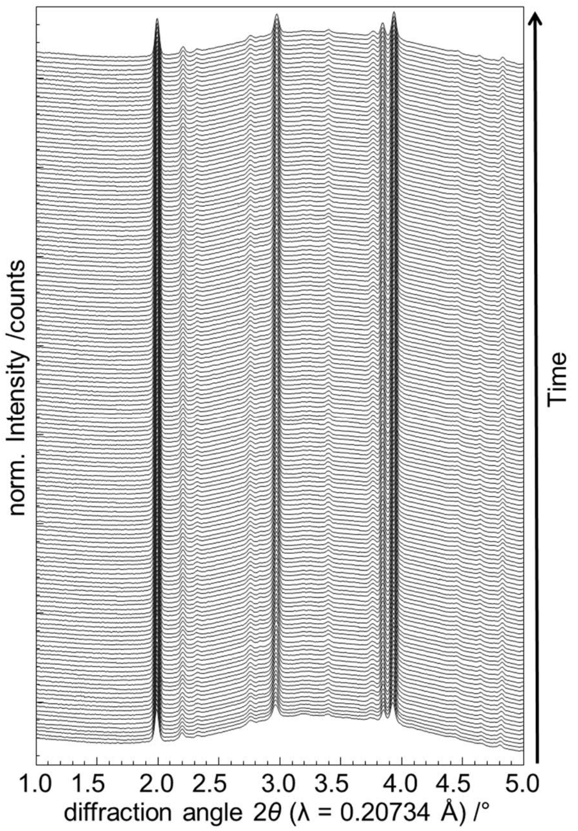

Figure S4. *In situ* time-dependent synchrotron XRPD data from the holding sequence of 30 min of the experiment TiP-1 (Table 1; reaction mixture TiO_2 : $NH_4H_2PO_2$ 1 : 6).

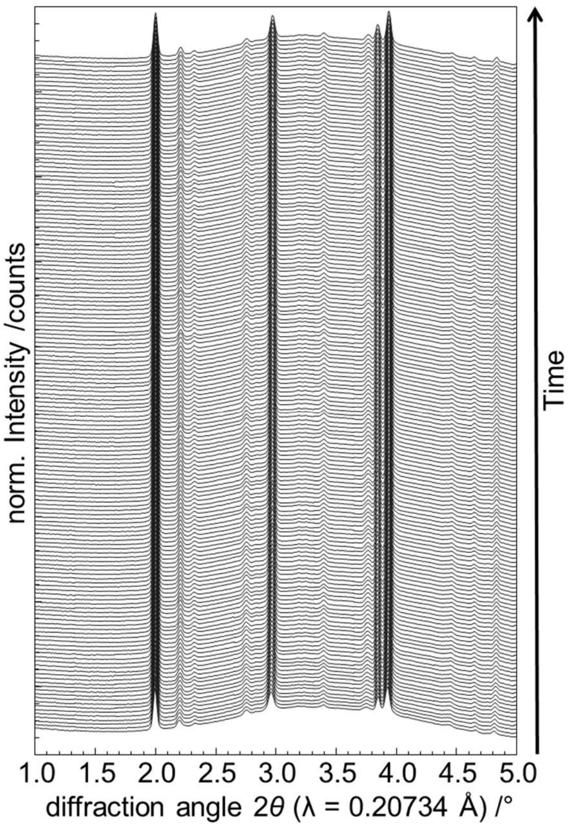

Figure S5. *In situ* time-dependent synchrotron XRPD data from the holding sequence of 30 min of the experiment TiP-3 (Table 1; reaction mixture TiO_2 : $NH_4H_2PO_2$ 1 : 8).

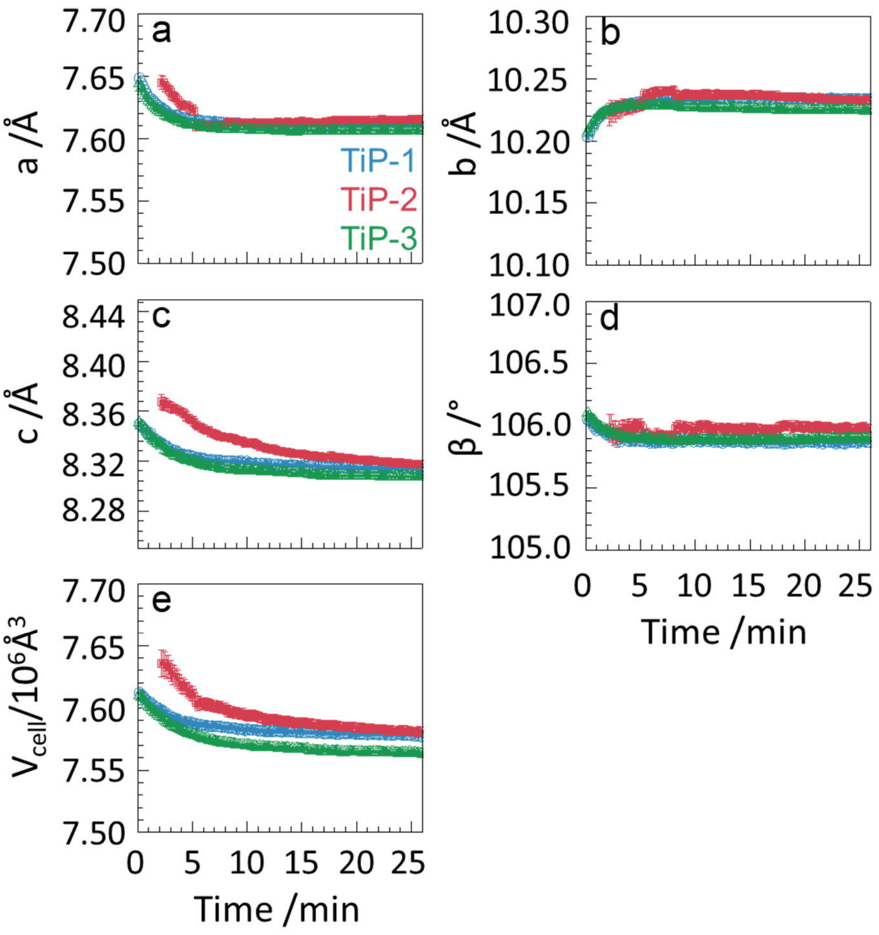

Figure S6. Selected time-dependent structural parameters of **Ti(III)p** determined during the holding sequence of 30 min of the *in situ* experiments TiP-1 (blue), TiP-2 (red), and TiP-3 (green): (a) lattice parameter *a*, (b) lattice parameter b, (c) lattice parameter *c*, (d) angle *β*, and (e) cell volume.

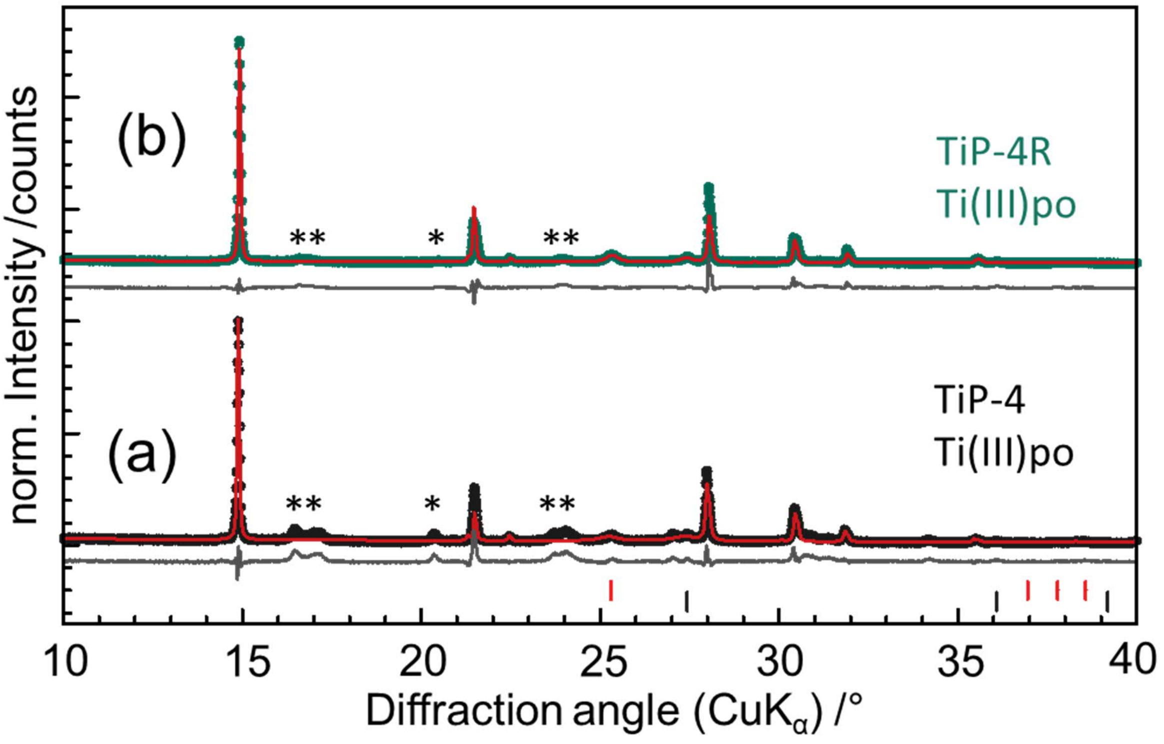

Figure S7. Rietveld plots of the ex-situ synthesis experiments testing the reproducibility to form **Ti(III)po** (Table 1). (a) Sample TiP-4 and (b) TiP-4R data points represent the XRPD data of the two synthesis products after washing to neutrality. The red line represents the calculated data from the respective model. The difference curve is shown in grey. The reflections of impurities are marked in the following manner: anatase (red bar), rutile (black bar), polyphosphate (asterisks).

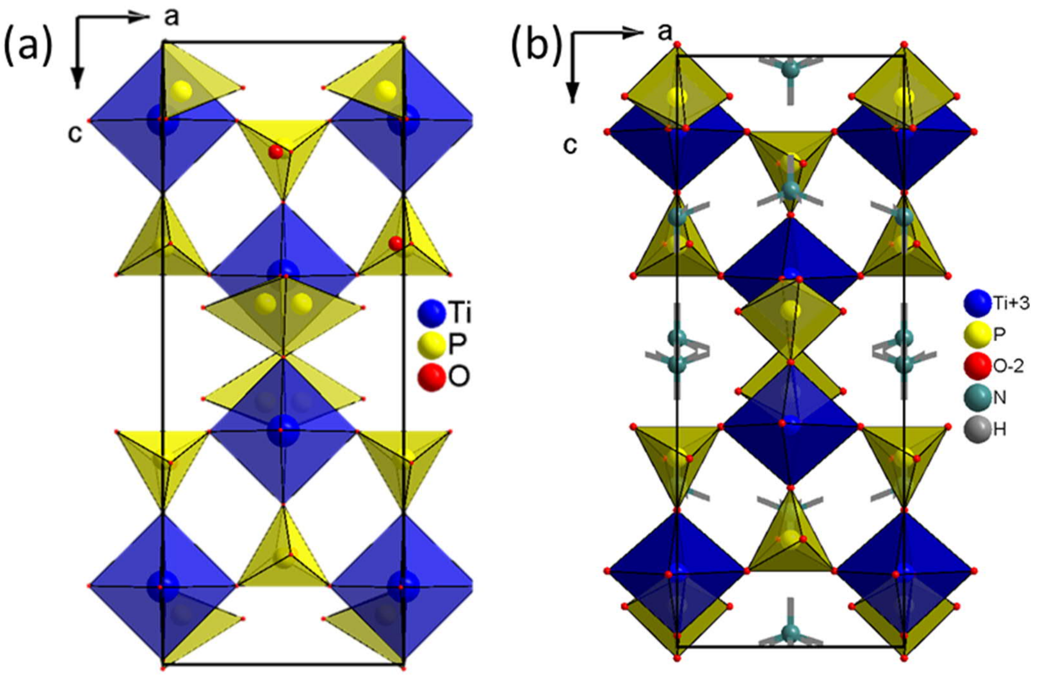

Figure S8. Crystal structures of (a)**Ti(IV)(HPO_4)$_2$** and (b)**Ti(III)po** with the chemical composition $(NH_4)_xH_{1-x}Ti(HPO_4)_2$.

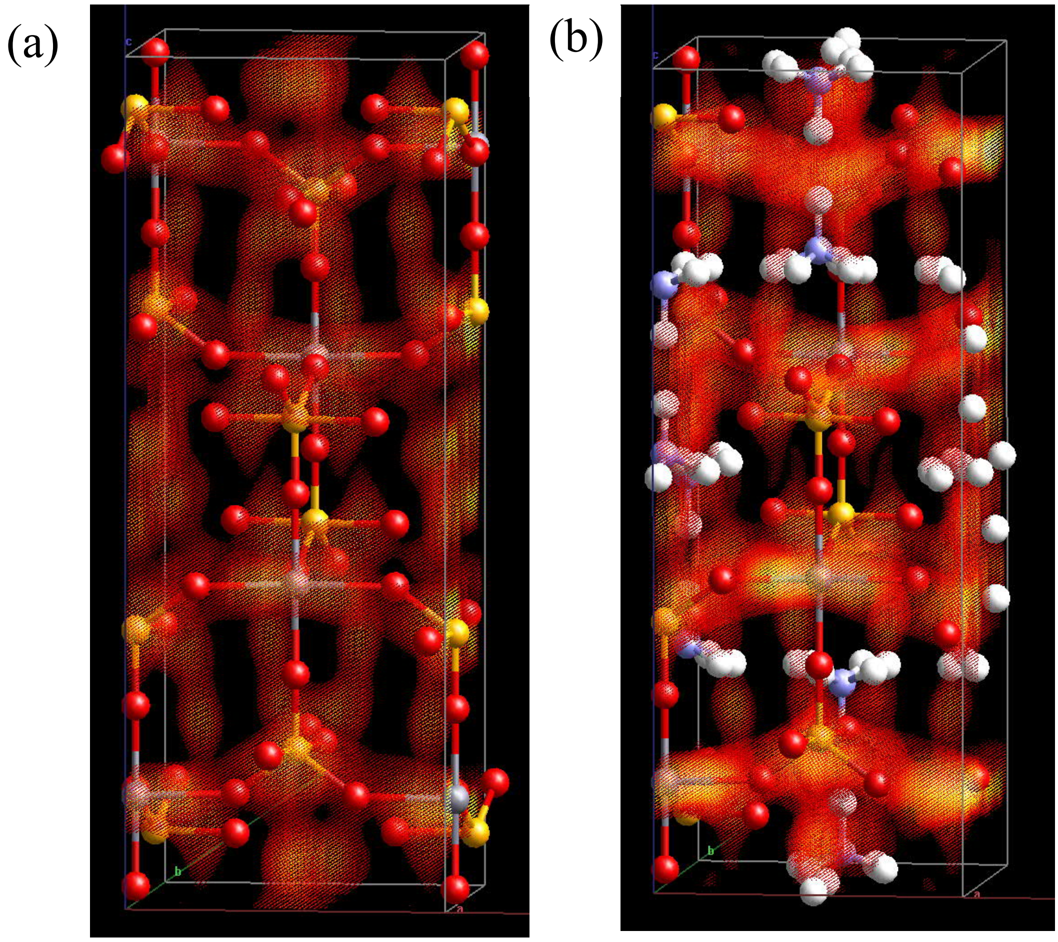

Figure S9. Difference Fourier analysis of **Ti(III)po**: (a) without considering NH_4^+ cations and (b) with implementing the cations.

Table S1. Crystal structure data of **Ti(III)po**.

$(NH_4)_xH_{1-x}Ti(HPO_4)_2$ (with x=0.41)						
Tetragonal, $I4_1/a$ (88)						
M ($gmol^{-1}$)	493.7					
a (Å)<br>b (Å)<br>c (Å)	6.3585(1)<br>6.3585(1)<br>16.5377(6)					
V ($Å^3$)	668.63(4)					
Z:	2					
R_{wp} (%)	12.2					
Site	Wyckoff position	x	y	z	occupancy	B_{iso} /$Å^2$
Ti1	4*a*	0	0.25	0.125	1	1
P1	8*e*	0.5	0.25	0.1805(7)	1	1
O1	16*f*	0.307(3)	0.288(5)	0.128(1)	1	1
O2	8*e*	0	0.25	0.231(2)	1	1
O3	16*f*	0.445(9)	0.019(2)	0.182(2)	0.5	1
N	8*e*	0	0.75	0.27236	0.205	1
H1	16*f*	0.000(5)	0.747(5)	0.333(5)	0.205	1
H2	16*f*	-0.146(5)	0.748(5)	0.250(5)	0.205	1
H3	16*f*	0.047(5)	0.893(5)	0.254(5)	0.205	1
H4	16*f*	0.117(5)	0.657(5)	0.254(5)	0.205	1

Table S2. Selected interatomic distances and angles of **Ti(III)po**.

Bond	Bond length /Å	Bond	Bond length /Å	Angle	Angle /°
P1-O1	1.522(1)	Ti-O1	1.970(1)	O1TiO1	90.03(2)
P1-O2	1.465(1)	Ti-O2	1.752(1)	O1TiO2	91.40(2)
P1-O3[a]	1.51(1)			O1TiO2	88.60(2)
				O2TiO2[b]	180
				O1P1O1	110.25(1)
				O1P1O20	124.88(1)
				O1P1O3	92.2(2)
				O2P1O3	89.13(1)

[a] Oxygen from the hydroxyl group

[b] axial oxygen from the TiO_6 octrahedron

Table S3: Crystal structure data of **H-Ti(III)po**.

$(NH_4)_xH_{1-x}Ti(HPO_4)_2$ (with x =0)						
Tetragonal, $I4_1/a$ (88)						
M ($gmol^{-1}$)	239					
a (Å)<br>b (Å)<br>c (Å)	6.3262(2)<br>6.3262(2)<br>16.2751(8)					
V ($Å^3$)	651.34(7)					
Z:	4					
R_{wp} (%)	13.6					
site	Wyckoff position	x	y	z	occupancy	Biso /$Å^2$
Ti1	4*a*	0	0.25	0.125	1	1
P1	16*f*	0.481(5)	0.297(5)	0.176(1)	0.5	1
O1	16*f*	0.25(1)	0.21(2)	0.160(3)	0.5	1
O2	16*f*	0.623(7)	0.25(1)	0.099(2)	0.5	1
O3	16*f*	0.47(2)	0.544(5)	0.174(3)	0.5	1
O4	16*f*	0.54(1)	0.26(1)	0.267(1)	0.5	1

Table S4: Selected interatomic distances and angles of **H-Ti(III)po**.

Bond	Bond length /Å	Bond	Bond length /Å	Angle	Angle /°
P1-O(1-4)	1.562(5)	Ti-O1	1.73(1)	O1TiO1	96(1)
		Ti-O4	1.78(1)	O1TiO4	106(4)
				O1TiO4	62(3)
				O4TiO4b	167(4)
				O14P1O1-4	109.28

[a] Oxygen from the hydroxyl group

[b] axial oxygen from the TiO_6 octrahedron
